# High-Salt-Diet (HSD) aggravates the progression of Inflammatory Bowel Disease (IBD) via regulating epithelial necroptosis

**DOI:** 10.1186/s43556-023-00135-1

**Published:** 2023-09-11

**Authors:** Jialong Qi, Jinli Wang, Ying Zhang, Huan Long, Liang Dong, Ping Wan, Zan Zuo, Wenjie Chen, Zhengji Song

**Affiliations:** 1 Department of Gastroenterology, Yunnan Digestive Endoscopy Clinical Medical Center, The First People’s Hospital of Yunnan Province, Kunming, 650032 P.R. China; 2 Yunnan Provincial Key Laboratory of Clinical Virology, The First People’s Hospital of Yunnan Province, Kunming, 650032 P.R. China; 3 School of Medicine, Kunming University of Science and Technology, Affiliated By The First People’s Hospital of Yunnan Province, Kunming, 650504 Yunnan P.R. China; 4 State Key Laboratory of Respiratory Disease, Guangdong-Hongkong-Macao Joint Laboratory of Respiratory Infectious Disease, Guangzhou Medical University, Guangzhou, 510182 P.R. China; 5 Sydney Vital Translational Cancer Research Centre, Westbourne St, Sydney, NSW 2065 Australia

**Keywords:** IBD, HSD, Necroptosis, RIPK3

## Abstract

Due to its unclear etiology, there is no specific medicine to cure the recurrent and incurable inflammatory bowel disease (IBD). Unhealthy dietary habits unconsciously contributed to the progression of IBD, for example a High-Salt-Diet (HSD) is the most neglected and frequently adopted habit. However, the molecular mechanism of how HSD aggravates the progression of IBD has yet to remain uncovered. Herein, we focus on the hypothesis that necroptosis pathway may be involved in the process of IBD exacerbated by HSD. To this end, different gene expression (DEGs) profiles of human epithelia under hypertonic culture conditions were applied to screen candidate pathways. What’s more, gene expression manipulation, immune microenvironment detection, RIPK3/MLKL gene knockout (KO), and wild-type (WT) mice were carried out to research the promotion of IBD progression under treatments of high salt intake. Based on our present results, gene expression profiles in human normal colon epithelia cell NCM460 were significantly changed under salt- or sucrose-induced hypertonic culture conditions. RIPK3 was significantly up-regulated under both conditions. Furthermore, mice colon epithelia cell CT26 growth was inhibited in a time- and dose-dependent manner by extra NaCl incubation. Autophagy, and Necroptosis pathways were activated and enhanced by LPS pretreatment. HSD significantly exacerbated DSS-induced IBD symptoms in vivo in a dose-dependent manner. Moreover, RIPK^3-/-^ and MLKL^-/-^ mice presented severe IBD symptoms in vivo. Overall, the results demonstrated that HSD aggravated the IBD progression via necroptosis activation, providing novel strategies and promising targets for the clinical treatment of IBD.

**Supplementary Information:**

The online version contains supplementary material available at 10.1186/s43556-023-00135-1.

## Highlights

1. High-salt and high-sucrose incubation enhanced RIPK3-dependent necroptosis in colonic epithelial cell.

2. HSD promotes DSS-induced IBD progression in a dose-dependent manner.

3. RIPK3 and MLKL genetic deletion significantly susceptible to IBD process.

**Supplementary Information:**

The online version contains supplementary material available at 10.1186/s43556-023-00135-1.

## Introduction

IBD is an idiopathic intestinal inflammatory disease at ileum, colon, and rectum tissues. It encompasses two subtypes, ulcerative colitis (UC) and Crohn’s disease (CD) [1]. Global epidemiological statistics indicate a continuous increase incidence of IBD continued to increase in both developed and developing countries [2]. Until now, immune factors [3], genetic factors [4], environmental factors [5], gut microbiome [6], and poor dietary habits [7] have been identified as factors associated with the onset of IBD. Although UC seems common in the adult and the elderly, in fact, it could develop at any ages, even in very early childhood [8]. Moreover, with the rapid development of UC diagnosis technology, over 25% of patients have been diagnosed with UC before the age of 18, resulting in rapid disease progression and the manifestation of and more severe phenotypes [9]. However, IBD remains incurable. Therefore, it is essential to comprehend its pathophysiology and develop innovative therapies for IBD patients.

Salt is a critical condiments in cooking, plays a critical role in maintaining the osmotic pressure stability of body fluids [10]. Excessive intake of salt also can perform as the “silent killer” attacking health by developing hypertension and cardiovascular diseases [11]. HSD has emerged as the most prevalent and underestimated dietary risk factor for various human diseases, exhibiting distinct geographical distribution, especially in East Asia. According to WHO’s (Word Health Organization) health report, the daily salt intake of East Asian residents was double the recommended dosage [12]. HSD has been demonstrated to significantly exacerbate the colitis process induced by DSS (Dextran sulfate sodium) via lactobacillus and butyrate production [13]. Moreover, recent studies revealed the double-sword role of HSD in IBD progression. Mice pre-fed with HSD were found elevated levels of IL-17A-producing T cells in lamina propria (LP), thereby increasing the susceptibility to TNBS (2,4,6-Trinitrobenzene sulfonic acid)-induced CD progression [14]. Conversely, HSD significantly reduced IL10-/- triggered IBD via promoting intestinal epithelial integrity and decreasing inflammatory cytokines release [15]. Nevertheless, the molecular mechanism of intestinal cell death in HSD-induced damage remains unclear.

Necroptosis is a classic programmed cell death (PCD) that is triggered by damage-associated molecular patterns (DAMPs) and pathogen-associated molecular patterns (PAMPs) stimulus. Upon binding of DAMPs or PAMPs, the signaling cascade is activated, leading to the recruitment of RIPK1-RIPK3-MLKL-necrosome complex. resulting in MLKL phosphorylation and oligomerization to form a pore in the cell membrane [16]. The activation of necroptosis has been implicated in various diseases, such as cancer, pathogen infection, nerve damage, and IBD [17]. In vivo studies have shown that the RIPK1 inhibitor Nec-1, RIPK3 inhibitor GSK’872, and MLKL inhibitor Necrosulfonamide (NSA) significantly reduced the severity of IBD. Moreover, the novel pan-necroptosis inhibitor LY3009120, screened from 611 small molecules has demonstrated a protective effect in DSS-induced IBD [18]. Additionally, genetic deletion of RIPK1 in vivo triggered Z-conformation nucleic acid binding protein 1 (ZBP1)-dependent necroptosis and inflammatory activation, RIPK3-/- mice showed decreasing Colorectal cancer (CRC) via c-Jun N-terminal kinase (JNK) pathway and CXCL1 (C-X-C Motif Chemokine Ligand 1) pathways, and MLKL-/- mice were susceptible to DSS-colitis and AOM-DSS-CRC via MEK/ERK pathway [19]. Collectively, these studies demonstrated the potential role of necroptosis in IBD progression.

Herein, we showed, for the first time, that HSD exacerbated DSS-induced colitis phenotype in a necroptosis-dependent manner. The exposure to salt or sucrose significantly triggered human epithelial cell death and altered gene expression profiles in vitro. The necroptosis adaptor RIPK3 was up-regulated in cells treated with either salt or sucrose. Similar results were obtained in murine epithelial cells, indicating that RIPK3 was also activated in a time- and dose-dependent manner following exposure to high salt treatment. Genetic deletion of RIPK3 or MLKL was susceptible to DSS-induced colitis in vivo. Taken together, our results elucidated a novel molecular mechanism responsible for the development of HSD-induced colitis.

## Results

### High-throughput sequencing revealed RIPK3 activation in NCM460 under high salt and sucrose condition

Maintaining an appropriate osmotic environment plays a pivotal role in human health. It is important to note that high-salt-induced hypertonic stress not only causes cardiovascular disease but also serves as a trigger for the onset of the IBD [11]. In order to elucidate the molecular mechanism caused by hypertonic stress on colon epithelia, we performed human epithelial cell line NCM460 cultured under hypertonic stress with 400 mM NaCl and 300 mM sucrose treatment. Subsequent to treatment, the total cell RNA was extracted and subjected to high-throughput sequencing assay. All data of each sample were collected no less than 10.78 G of valid data base, with a high Q30 percent and GC content percent, indicating high quality of data. Furthermore, more than 63% of the mapping region was identified in the exon region for all samples (Supplementary Fig. 1). The QC data and mapping data was presented in Supplementary Tables 1 and 2.

**Fig. 1:**
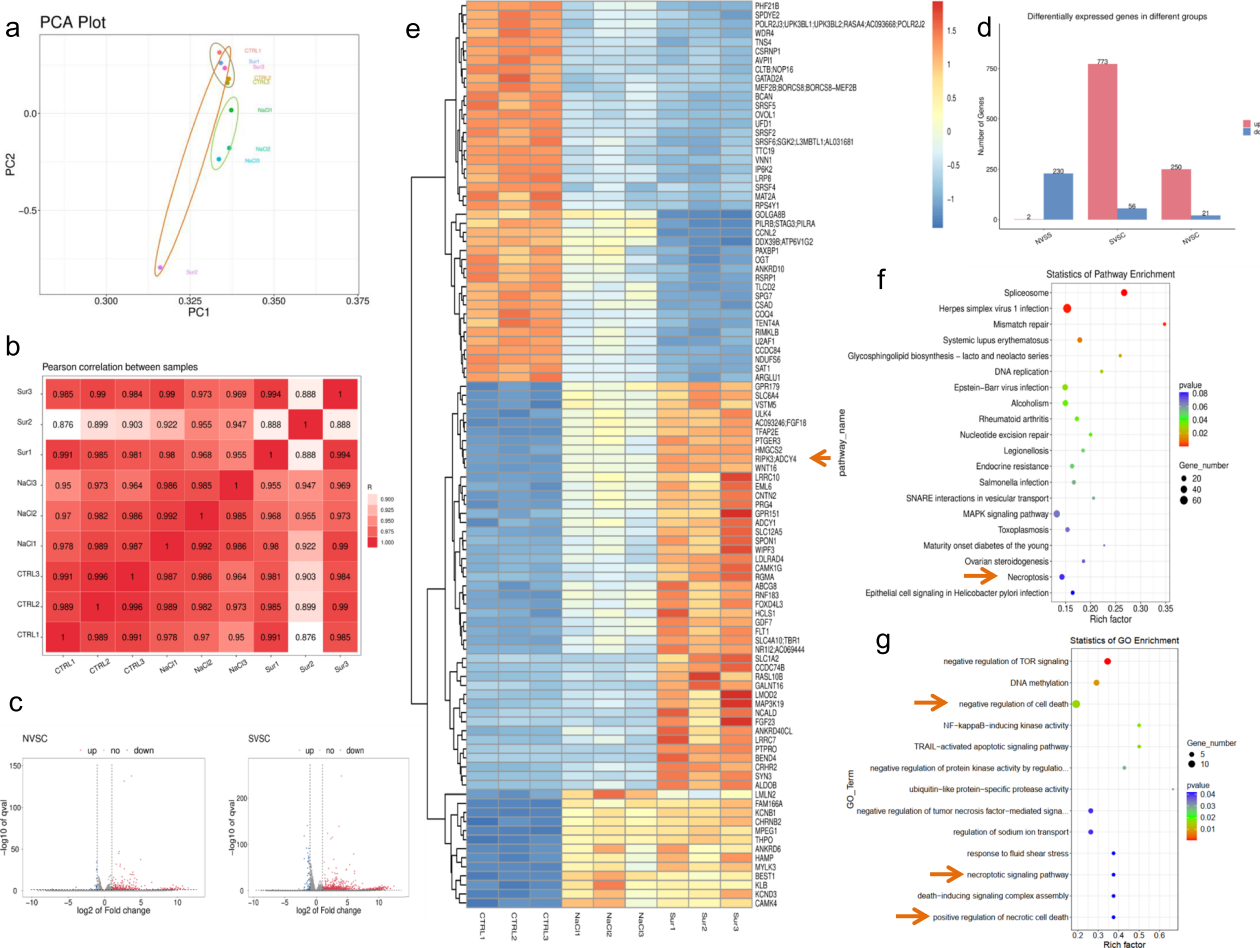
Necroptosis-related signaling pathway was activated in high-salt- or sucrose- incubated human epithelial cell. **a** PCA plots of high-salt and high-sucrose treated cells. Three sample of each group were showed here. **b** Pearson correlation between all samples, CTRL represented control group; NaCl represented high-salt group; Sur represent high-sucrose group. **c** Volcano maps showed different expression genes (DEGs) between N vs C and S vs C. Blue plots represented down-regulated genes and red plots represented up-regulated genes. **d** Histogram showed the number of DEGs between two groups. **e** Heat map showed top 100 DEGs among three groups. Arrows showed RIPK3 was up-regulated in both high-salt/sucrose incubated cells. **f** KEGG enrichment of DEGs. **g**. GO analysis of DEGs. Arrows showed necroptosis-related signaling pathways were activated here

The gene expression of all samples showed no difference across all samples, as indicated by the log_10_ (FPKM), and the correlation cluster and PCA assay are shown in Supplementary Fig. 2. According to gene expression data, a total of 773 up-regulated and 56 down-regulated genes were in the sucrose-treated group compared with the Ctrl group, meanwhile, there were 250 up-regulated and 21 down-regulated genes in the NaCl-treated group (Fig. 1d). Volcano graph showed changed genes and the heatmap showed the top 100 changed genes (Fig. 1c,e). Notably, we found that RIPK1/3 was activated in both high-salt- and high-sucrose-treated groups (Fig. 1e). Moreover, the GO and KEGG databases were applied for function pathway enrichment assay. We found that the necroptotic signaling pathway, positive regulation of necrotic cell death, regulation of sodium ion transport, and negative regulation of TNF-mediated signaling pathway were activated with GO enrichment, meanwhile, pathogen infection, Mitogen-activated protein kinase (MAPK) signaling pathway, and necroptosis were enriched via KEGG database (Fig. 1f-g). Thus, our results showed that high-salt or high-sucrose may trigger cell necroptosis activation. In the meantime, two groups revealed that high-salt-induced hypertonic stress-activated SLC9A1 (Solute Carrier Family 9 Member A1) resulting the activation of necroptosis in vitro and in vivo [20]. Altogether, these results reveal the role of necroptosis under high salt-induced hypertonic stress stimulation.

### High salt exposure triggered murine colon epithelia necroptosis

**Fig. 2:**
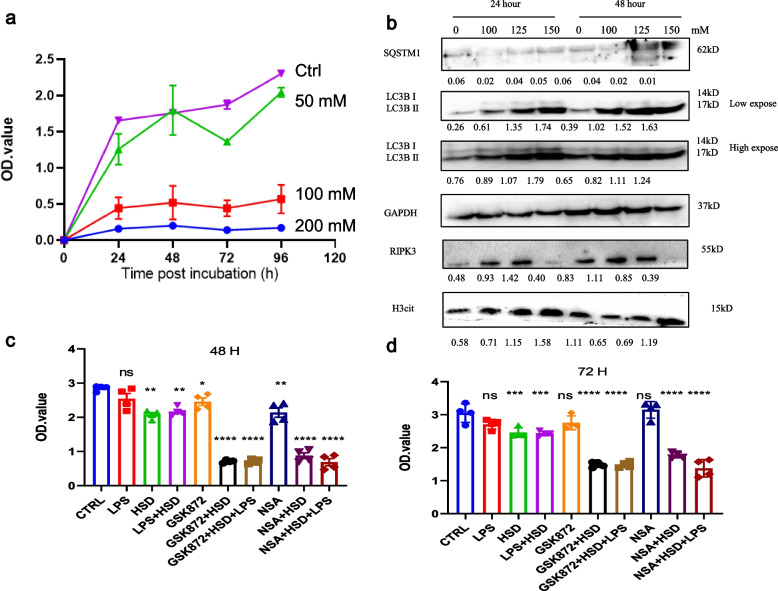
High-salt incubation suppressed cell growth and triggered necroptosis. **a** CCK-8 assay of CT26 cell incubated with extracellular salt. 1 × 10^5^ CT26 cells were treated by different NaCl concentrations which were 50, 100, 150, 200 mM for 24, 48, 72, and 96 h. **b** Western blotting analyzed PANoptosis cell marker expression level of HSD treated CT26. SQSTM1, LC3, RIPK3, and H3cit were showed here. CCK-8 assay of the role of necroptotic pathway inhibitors in HSD treated cells. **c** 48 h; **d**. 72 h. ** *p* < 0.01;*** *p* < 0.001; ****p < 0.0001

To more specifically elucidate the role of necroptosis in colitis, we also cultured murine colon epithelia CT26 with extra-salt in vitro. The cell growth curve revealed that HSD treatment significantly decreased cell viability in a time- and dose-dependent manner (Fig. 2a). Autophagy was one of the most important Programmed cell death (PCD) for maintaining cell stability under serval stress stimulation. Herein, we found that HSD treatment significantly promotes cell autophagy in a time and dose-dependent manner, and the ratio of LC3 II/LC3 I was increased. Meanwhile, the H3cit protein expression level was also up-regulated. The expression of RIPK3, the kinase of necroptosis, was also upregulated, reflecting necroptosis activation under HSD treatment (Fig. 2b). Moreover, RIPK3 and MLKL specific inhibitor GSK’872 and NSA significantly reduced cell viability under HSD treatment (Fig. 2c-d). Taken together, our results reveal that HSD treatment also activated necroptosis in murine colon epithelia.

### Pretreatment with LPS enhance cell necroptosis in vitro

**Fig. 3:**
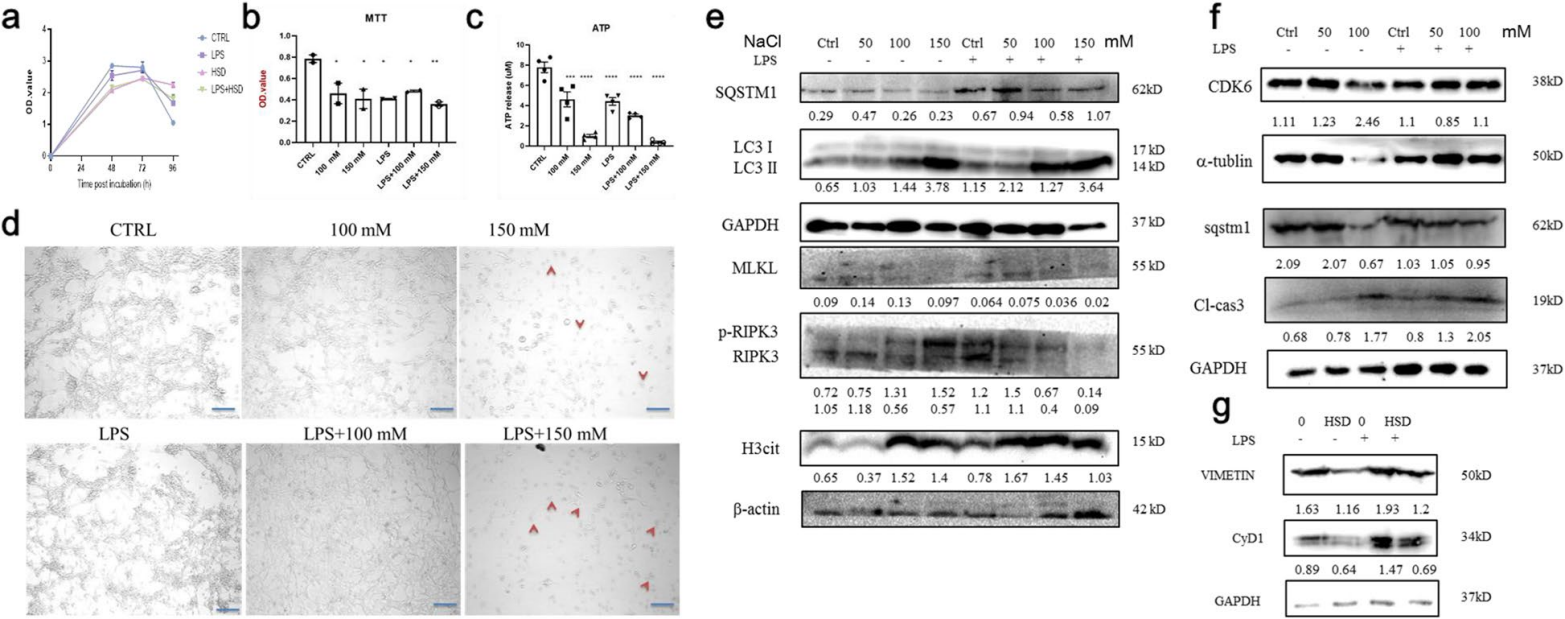
LPS pre-treatment enhanced HSD-induced cell death. **a** CCK8 analysis of CT26 growth curve. CT26 were treated by HSD (150 mM), LPS (100 ng/ul), LPS + HSD. **b** MTT assay showed LPS plus HSD treatment enhanced cell death in vitro. **c** ATP release assay of CT26 under different treatment. **d** Represent pictures of CT26 cell treated with HSD and/or LPS. **e**–**g** Western blotting analysis of CT26 cells under different treatments. * *p* < 0.05;** *p* < 0.01;*** *p* < 0.001; ****p < 0.0001

Gut microenvironment homeostasis was critically important for the progression of IBD. Extensive evidence revealed that gut microbiome and metabolites were altered in IBD patients [21]. Herein, we found that pretreatment of LPS, which is one of the most important cell-wall contents in Gram-negative bacteria, combined with high-salt treatment could significantly decrease cell growth (Fig. 3a). MTT detection indicated LPS enhanced cell death in vitro (Fig. 3b). And we also found that cellular ATP storage was decreased (Fig. 3c). Furthermore, LPS pre-treatment significantly enhanced autophagy, apoptosis, and necroptosis as well as induced cell cycle arrest (Fig. 3d-f). The expression level of SQSTM1 were decreased and LC3 II level were increased in a dose-dependent manner. The increased MLKL, RIPK3, cleaved Caspase 3 and H3cit expression levels revealed cell apoptosis and necroptosis were activated. Furthermore, we also found that Vimentin, CyD1 and CDK6 were decreased. Altogether, our results demonstrated LPS combine with HSD treatment induced colon epithelia cell death in vitro.

### DSS-induced IBD significantly reshaped immune microenvironment

**Fig. 4:**
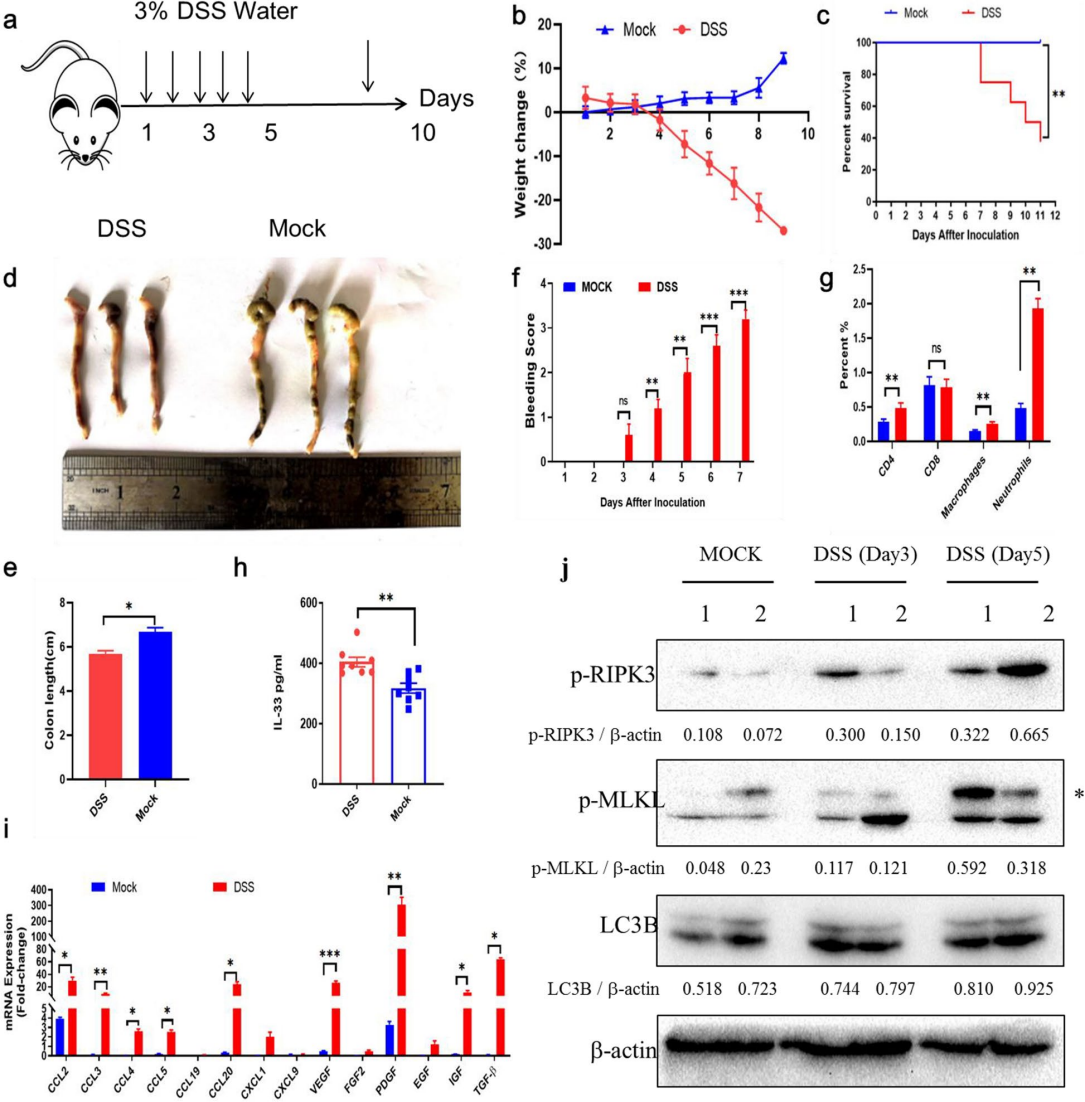
Necroptosis was activated in DSS-induced colitis. **a** Schematic diagram of DSS-induced colitis model. C57 mice were fed with 3% DSS-water for 7 days and sacrificed on day 12. **b** Weight change of mice in Mock and DSS group (*n* = 8). Mice weights were monitored every day. **c** Survival curves of mice in Mock and DSS group (*n* = 8). **d** Represent pictures of colon tissues. **e** Histogram showed colon length between Mock and DSS group. **f** Anus bleeding score. **g** Flow cytometry assay of splenic lymphocyte between Mock and DSS group. CD4, CD8, macrophages, and neutrophils were showed here. **h** Serum IL-33 level between Mock and DSS group (*n* = 8). **i** RT-PCR results showed cytokines and chemokines between Mock and DSS group. **j** Western blotting of LC3B, MLKL and RIPK3 level in colon tissues. * *p* < 0.05;** *p* < 0.01;*** *p* < 0.001

Although our previous results indicated that HSD triggered human and murine colon epithelial necroptosis in vitro, the role of HSD-induced necroptosis in IBD still remained unclear. To this end, we first constructed a DSS-induced colitis model (Fig. 4a). After 5 days incubation with water containing 3% DSS, the weight of mice was significantly decreased, disease score and fecal blood were increased (Fig. 4b-f). For the evaluation of collected colon tissues, the colons in the DSS water-treated group were shorter than those of control group (Fig. 4e). In the meantime, we found that spleen lymphocytes components were also reshaped, percentages of CD4 cells, macrophages, and neutrophil were significantly increased (Fig. 4g). IL33-ST2-axis also showed potential role in IBD, we also detected IL33 concentration in serum that was increased in DSS group (Fig. 4h). The secretion of inflammatory cytokines in colon tissues like ccl2/3/4/5/20, VEGF/PDFG, and IGF/TGF-β were enhanced (Fig. 4i). Most importantly, we found that colon necroptosis pathway was also activated (Fig. 4j). Thus, we found that the immune environment was altered in IBD model and trigger colon necroptosis activation.

### Pre-treat with HSD enhanced IBD in vivo

**Fig. 5:**
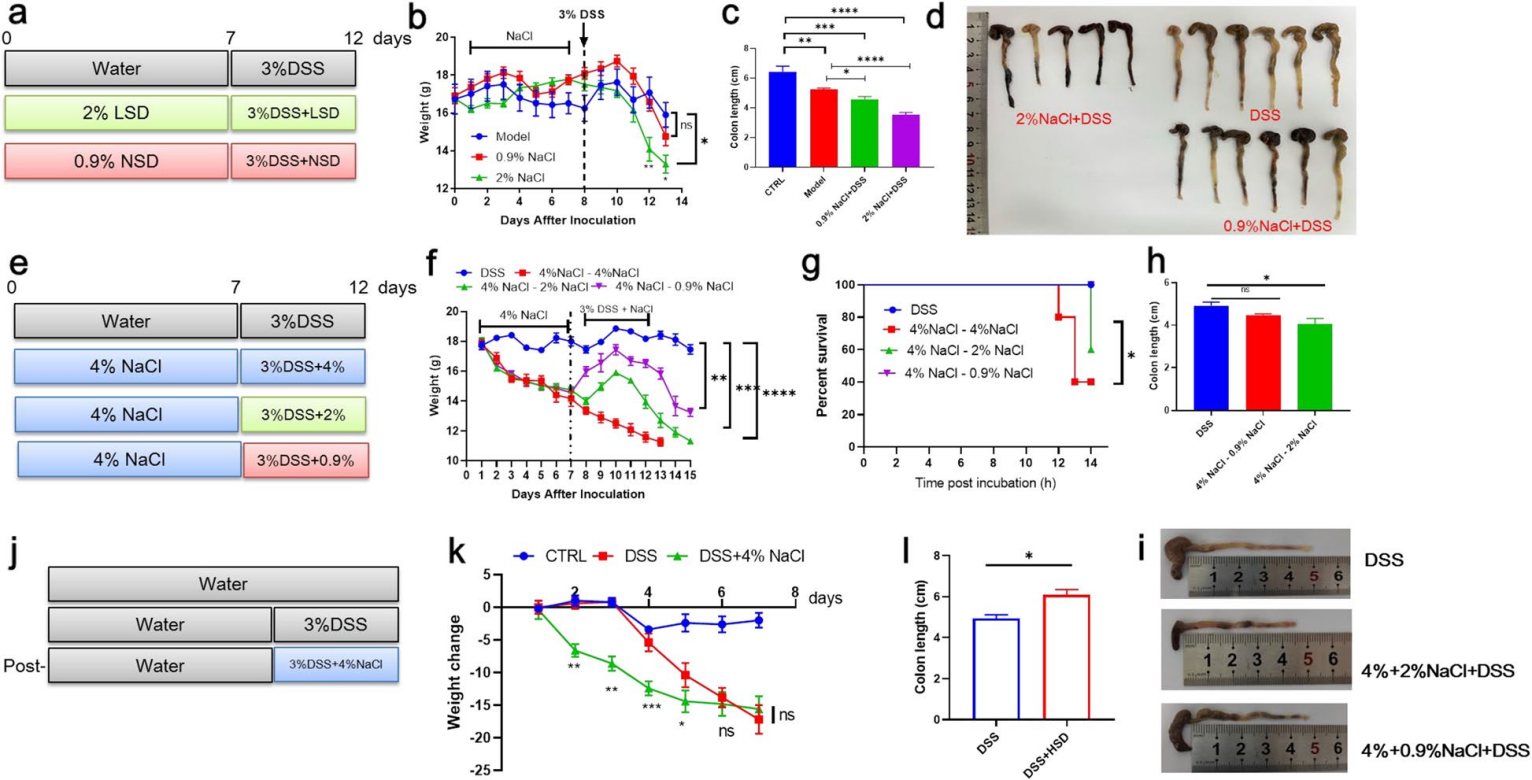
HSD increased IBD process in vivo. **a** Schematic diagram of salt concentration on IBD progression. Mice were treated with 2%, 0.9%, and 0% NaCl-H_2_O for a week and then fed with 3% DSS, (*n* = 6). **b** Mice weight change curve of each group. **c** Colon length of each group. one mice in 2% NaCl group were dead before the endpoint. **d** Represent picture of colon tissues of each group. **e** Schematic diagram of salt change in IBD process. **f** Mice weight change curve of each group. **g** Survival curve of each group. **h** Colon length of each group. **i** Schematic diagram of post-HSD treatment on IBD process. **j** Schematic diagram of salt concentration on IBD progression. **k** Weight change of each mice. **l** Colon length of DSS and DSS-HSD group. * *p* < 0.05;** *p* < 0.01;*** *p* < 0.001; ****p < 0.0001

To investigate the role of HSD on IBD progression, we performed an HSD-IBD model here. Firstly, we screen the effect of mice fed with different salt concentrations, all mice were dead in the first week at the 8% NaCl group, and there was a fluctuation revert in 4% and 2% groups, while there was no difference between 0.9% and CTRL group (Supplementary Fig. 3). To demonstrate the role of salt concentration on IBD, mice were pretreated with a gradient dose of NaCl for a week and then fed with DSS (Fig. 5a). Food/water intake, disease score, and blooding were recorded every two days, As shown in Fig. 5a, the disease server was performed in a dose-dependent manner and the colon tissues were collected at the endpoint, the colons were shorter in a higher dose and more blooding occurred (Fig. 5b-d). Then, we analyzed the role of salt change on IBD progress. Mice were first given a 4% high dose for a week and then changed to a lower dose. As shown in Fig. 5e, the decrease of salt significantly suppressed IBD progression which was reflected in the weight coverage, and colon length (Fig. 5f-i). At last, we analyzed the direct influence of HSD and DSS on IBD (Fig. 5j). Interestingly, although the weight decreased more quickly than DSS group, the average colon length was longer than that of mice which is caused by the water intake decreasing in HSD group (Fig. 5k-l). Altogether, our HSD-IBD model revealed that HSD treatment significantly promoted IBD progression in vivo.

### RIPK3^-/-^ and MLKL^-/-^ was susceptible to DSS-induced colitis

**Fig. 6:**
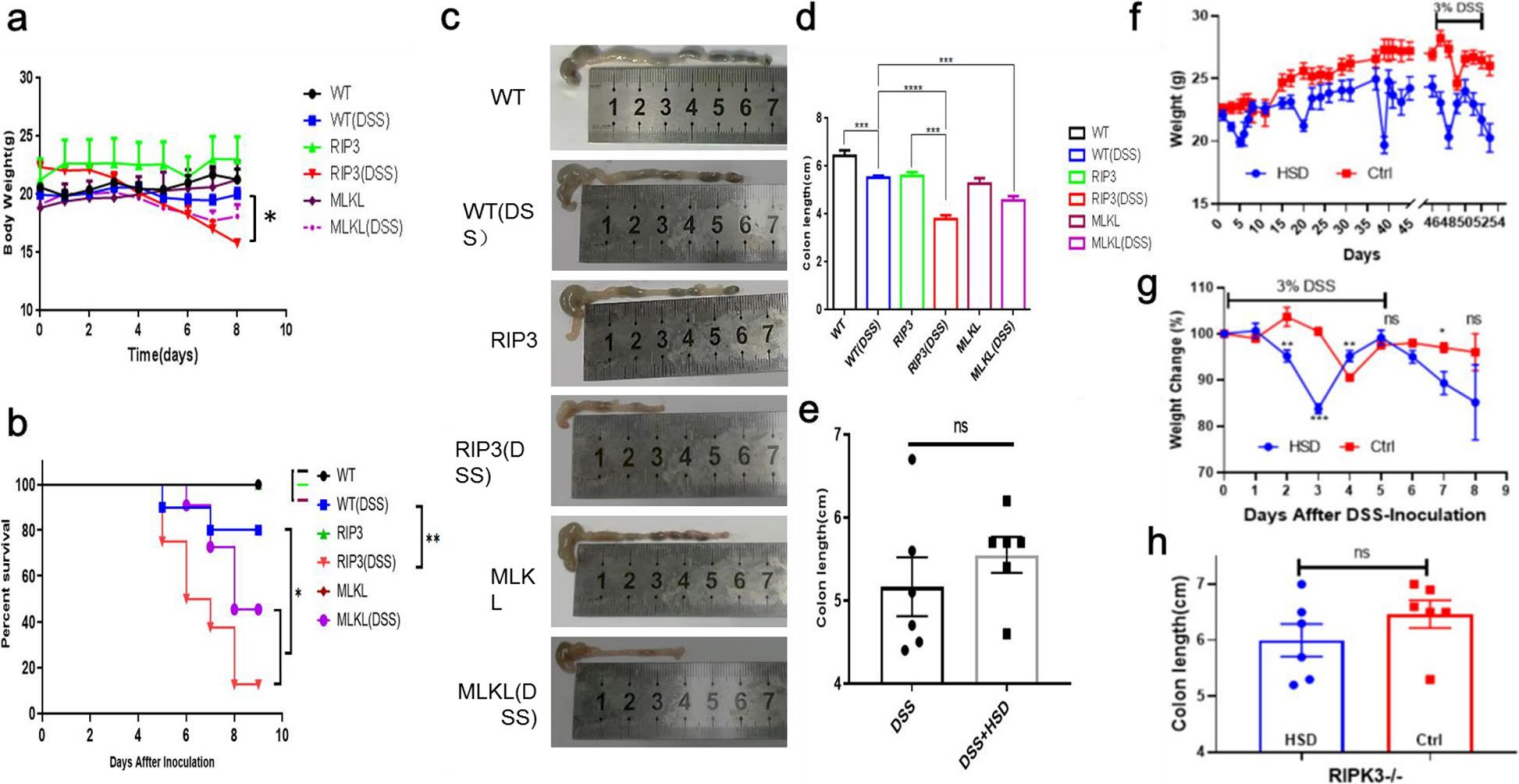
MLKL and RIPK3 KO mice showed susceptible to IBD process. **a** Weight change all mice treated with DSS or not. WT, RIP3 means RIPK3^-/-^, and MLKL means MLKL^-/^^-^. **b** Survival curve of WT and KO mice treated with DSS. **c** Represent colon tissues of each group. **d** Colon length of each group. **e** Colon length of RIPK3^-/-^ mice treated with DSS and DSS + HSD. **f** mice fed with HSD reduced weight and increased sensitivity to DSS. **g** Mice weight change curve. **h** Colon length in RIPK3^-/-^ fed with HSD or ND. *t*-test was applied for two groups comparison, one-way annoy was used for three or more groups comparison, and two-way annoy. * *p* < 0.05;** *p* < 0.01;*** *p* < 0.001; ****p < 0.0001

To specifically demonstrate the role of RIPK3 and MLKL in DSS-induced colitis, age-matched RIPK3^-/^- and MLKL^-/^- mice were applied here to compare with WT mice under DSS-treated. After 5 days of 3% DSS-water treatment, the weight of mice in the KO and WT groups were recorded daily. The weight loss in RIPK3^-/-^ and MLKL^-/-^ KO groups was more than that in the WT group (Fig. 6a). In the meantime, the survival curve demonstrated that RIPK3^-/-^ and MLKL^-/-^ mice were susceptible to DSS-induced colitis (Fig. 6b). At the experiment endpoint, mice colon tissues were collected and the represent pictures of colon tissues were shown in Fig. 6c. There was a significant difference among WT and RIPK3/MLKL KO mice (Fig. 6d). Moreover, we found that the knocking out of RIPK3 had no difference between DSS and HSD + DSS treatment indicating that necroptosis-related pathway was important for HSD induced colitis (Fig. 6e). We also fed mice with HSD and normal diet for 45 days and then treated them with DSS, mice weight in HSD group was more lighter than normal diet and the loss of weight was more quickly (Fig. 6f). However, This phenomenon did not occur in RIPK3 genetic mice (Fig. 6g). And the colon length showed no difference (Fig 6h).Taken together, our results demonstrated that RIPK3/MLKL KO mice were susceptible to DSS-induced colitis.

## Discussion

In this study, we primarily identified the new molecular mechanism of HSD-induced IBD which is highly associated with necroptosis. Moreover, through high-throughput mRNA sequencing, we further revealed that the RIPK3-dependent necroptosis pathway was activated in human colon epithelia cells following exposure to high salt or sucrose incubation. Furthermore, high-salt stimulation inhibited cell growth of murine colon epithelia and triggered cell death in a time- and dose-dependent manner that was enhanced by LPS pre-treatment. Moreover, HSD was shown to significantly promot the IBD process in vivo and mice with genetic deletion of RIPK3/MLKL was susceptible to DSS-induced colitis.

Ulcerative colitis (UC) and Crohn’s disease (CD) are the two different clinical subtypes of inflammatory bowel disease (IBD) [22], with increasing incidence significantly impacting the quality of life for affected individuals. However, the behind pathogenesis is not completely validated. In general, immune factors, genetic factors, environmental factors, and bad diet habits all contribute to the IBD process [23]. Notably, a majority of the identified genetic susceptibility genes were related to the recognition and processing of microbial antigens on the surface of the intestinal mucosa. Our daily lifestyle renders us be prone to develop poor diet habits such as excessive intake of high-salt, high-fat, and high-sugar [1]. Multiple pieces of evidence indicated that above diet habits increased the risk of IBD, with the consumption of a high-salt diet being particularly detrimental and associated with the highest risk of developing the condition. HSD was almost involved in all processes that regulated IBD progression including gut microbiota, immune cell infiltration, and cytokines releasement [24]. A previous study revealed that HSD reshaped gut microbiota and bile acid homeostasis in IBD. Furthermore, HSD affects the interaction between the immune system and the gut microbiome, leading to a reduction of short-chain fatty acids (SCFA) like butyrate but also decreased the ratio of *Firmicutes* and *Bacteroidetes* as well as Lactobacillus [13]. Moreover, HSD activated TH17 cells, which contribute to a pro-inflammatory intestinal immune response. This activation occurs through the involvement of salt-sensing kinase SGK1 (Serum/Glucocorticoid Regulated Kinase 1) [25]. What’s more, HSD modulates colon ILC3 function and exacerbates TNBS-induced colitis [26]. Taken together, these studies indicated that HSD played a critical role in IBD progression.

However, the limited understanding of the precise molecular mechanism of HSD-triggered cell death has spurred increasing efforts to investigate this phenomenon. For instance, a recent study indicated that the HSD-activated NLRP3-dependent pathway promoted the process of hypertension [27]. Similarly, another group revealed extracellular Na^+^ intake positive feedback Ca^2+^ influx that triggered mitochondria damage to release ROS (reactive oxygen species, ROS) and activated NLRP3 inflammasome [28]. Additionally, Wang’s group used whole genome CRISPR-Cas9 screening to reveal that colon epithelia necroptosis was activated by high salt treatment that was dependent on the function of SLC9A1 (Solute Carrier Family 9 Member A1), a proton pump located on the cell membrane. The uptake of extracellular Na^+^ resulted in the exchange of H^+^ ions, causing an increase in intracellular pH and activated necroptosis [20]. Herein, we initially analyzed the role of high salt/sucrose treatment in human colon epithelia cells. Intriguingly, we found that the necroptosis-related pathway but not the pyroptosis-related pathway was significantly enriched via GO/KEGG assay. Consistent with the sequencing results, necroptosis-related pathways were activated in both treatments. We speculate that the difference here was due to the disease model and cell type. Our research, along with Wang's research, specifically focus on the development of intestinal disease but not cardiovascular disease. Notably, the necroptosis-related genes were over-expressed in intestinal cells that quickly reflected the response to DAMPs stimulation but not NLRP3-inflmmasome activation. Consequently, we further applied murine colon epithelia cells to screen the key molecular pathways under high-salt/sugar stress. In conclusion, we confirmed that high salt significantly activated the necroptosis signaling pathway in human colon epithelia which was also confirmed in a murine cell line.

Immune cell infiltration was also one of the key indicators for IBD processing [3]. In the last decade, extensive research revealed the connection with Th17 cells, a main subtype of CD4^+^ T lymphocytes which was involved in the pathogenesis of most autoimmune diseases, and IBD [29]. The production of IL-17A/F, IL-10, and TNF-α from mature Th17 cells shapes a pro-inflammatory environment for colon epithelia damage and altered microbiome and metabolite profiles [3]. Furthermore, the balance of Th17 and other cells like Th1, Th2, and Treg was also important for IBD patients [30]. Neutrophils work as the first defense line in host for anti-pathogen infection as well as autoimmune diseases like IBD. The ratio of neutrophils/lymphocytes was considered an IBD diagnosis biomarker [31]. Additionally, the crosstalk between neutrophils and T cells in intestinal tissues has been found confer benefits for IBD progress. Notably, Neutrophil extracellular traps (NETs) was also involved in the IBD process, the novel death of neutrophils which manifested as cell rupture, chromatin leakage, and activated macrophage type I interferon signaling pathway via cGAS-STING-dependent manner [32]. In our study, we found that splenic CD4 T cells, macrophages, and neutrophils were significantly increased in the DSS-treated group.

Pro-inflammatory cytokines released by epithelial cells and innate immune cells are key factors in the inflammatory reaction [33]. The alteration in the gut microbiome trigger fibroblast cells and enterocytes to release IL-33, resulting in the dysregulation of T cell microenvironments balance and intestinal tissue damage [34]. A recent study revealed that intestinal mesenchymal cells expressed IL-33 contributes to inflammation and intestinal barrier dysfunction in IBD. As for CD, the expression level of IL-33 is increased and correlated with CD disease progression. IL-33 has been reported to exacerbate TNBS-induced colitis by promoting a Th1 response. Meanwhile, in the SAMP1/YitFc spontaneous ileitis model, IL-33 was also involved in increased pro-fibrogenic Th2 response [35]. In a UC model, ST2-deficient mice displaced resistance to DSS-induced colitis, indicating that IL33-ST2-axis enhanced colonic mucosal healing. In addition, IL-33 activated the function of Th9 cells and ILC2s resulting in the disruption of intestinal barrier function [36]. Administration of exogenous murein-IL33 significantly abrogated epithelial damage, pro-inflammatory cytokine secretion, and loss of barrier integrity. Our result also confirmed that serum IL-33 releasement was upregulated in the DSS-induced IBD model. Importantly, we found that growth factors (GFs) like VEGF, IGF, and PDGF as well as chemo-cytokines CCL2, CCL3, CCL4, CCL5, and CCL20 were significantly upregulated in colitis’s colonic tissues. Angiogenesis has been reported closely associated with the initiation and development of chronic inflammation, which is not only correlated with the disease severity but also provided essential oxygen and nutrients for the migration of leukocytes to inflammation sites [37]. In addition, the expression of VEGF and MMP-9 promoted angiogenesis and enhanced vascular permeability resulting in the break of the immune barrier, interruption of immune-microbes interaction, and progression of IBD [38].

Necroptosis was a caspase-independent PCD that was involved in pathogen infection, cancer, and auto-inflammatory disease [16]. Necroptosis was a lytic cell death that was triggered by oligomerization of p-MLKL into a pore formation, which resulted in the release of inflammatory cytokines like IL-33, VEGF, CCL5 as well as MLKL, and RIPK3 to extracellular [17]. In clinical, the serum necroptosis-related protein level including RIPK1, RIPK3, and MLKL was also found increase in IBD patients. It has been reported that the expression level of RIPK3 and MLKL was increased in child-inflamed tissues of IBD and allergic colitis, which was also confirmed in the terminal ileum of adult CD patients [39]. And in the animal models, no matter in the DSS-induced UC model or TNBS-induced CD model, the intestinal mucosa expression of RIPK3 was increased, which was similar to human beings. A20-binding inhibitor of NF-κB activation protein 3 (ABIN3) negatively regulated inflammatory response in IBD via k63 de-ubiquitination of RIPK3 [40]. The autophagy-related ATG16L1 (Autophagy Related 16 Like 1) inhibited necroptosis in the epithelium in both animal model and human tissues that revealed autophagy and necroptosis interplay an important role in IBD [41]. Herein, we first found that HSD or HFD treatment significantly regulated necroptosis in vitro and that was also confirmed in the DSS-induced UC model.

Appropriate animal models allow for better understanding of the pathogenesis of IBD and then facilitate target drug screening and immune response monitoring [42]. Along with the developing process in genomic editing technology, multiple spontaneity IBD mice models had been developed such as IL10^-/-^, Mettl14^-/-^, and SETDB1^-/-^ [43]. However, compared with the spontaneous IBD model, the inductive model possessed advantages of genetic stability, conferring more efficient and reproducible studies. DSS-induced IBD mice model had been widely applied in multiple articles that were similar to UC in clinical, which performance weak with regard to T cell-based immune response [44]. With less investigation of the T cell-based immune response in this present work, we yet used the DSS-induced IBD model, focusing on HSD-induced epithelial cell damage in the IBD process. To more specifically revealed the role of necroptosis in HSD-induced IBD, genetic deletion of RIPK3/MLKL was constructed and applied here [45]. The KO mice showed sensitivity to DSS-induced colitis, further confirmed that bad diet HSD increased IBD progress via necroptosis.

## Conclusion

Herein, our work demonstrated a novel molecular mechanism for high salt-induced colitis via exploring the involvement and regulation by necroptosis signaling pathway. However, how HSD triggers necroptosis activation and the rescue role of SLC9A1 needs further investigation. In total, our research provided the potential role of necroptosis in IBD-induced colitis, which may supply new directions of targeting necroptosis for treating IBD.

## Methods and materials

### Ethic statement

Female C57BL/6 mice at six-to-eight-week were purchased from Beijing SiPeiFu animal technology co., maintained at Guangzhou Medical University under specific pathogen-free (SPF) conditions. Mice were subjected to adaptive feeding for 7 days before following treatment. All animal experiments were approved by SYXK (YUE) 2016–0168. Upon experiments completed, the remaining mice were suffocated to death in a closed carbon dioxide box after anesthesia. All efforts were made to minimize animal suffering.

### Animal model construction

The DSS-induced colitis model was widely applied as the UC model. Herein, mice were adaptive feed for a week and then divided into two groups according to the weight. The model group was fed with 3% DSS-water for 5 days while the control group was fed with normal water. The weight of mice, disease score, water, and food intake were monitored daily. At the experiment endpoint, colon tissues, serum, and spleen were collected.

### High-salt-diet (HSD) model construction

To analyze the role of high-salt-diet (HSD) in IBD process, extra NaCl was added to water. 0.9%, 2%, 4%, and 8% were first applied here to detect the survival rate of mice. We first evaluated the role of HSD on IBD process by analyzing different diet modes. First, the mice were fed with 4% NaCl for a week and then changed to 0%, 0.9%, 2% and 4% NaCl plus 3% DSS for 5 days. Then, mice were fed with 0%, 0.9%, 2% and 4% NaCl for 14 days and on day 8–12 mice were fed with extra 3% DSS. At last, mice were fed normally for a week, and then treated with 4% NaCl plus 3% DSS for 5 days.

### RT-PCR identification

Mice colon tissues were collected at the endpoint and total RNA was extracted in RNAzol®RT RNA isolation Reagent (GeneCopoeia, E01010A). All-in-one™ miRNA qRT-PCR detection kits (GeneCopoeia, AORT-0020) were applied here for specific gene detection. 2^−ΔΔt^ value was shown as gene fold change. The primers used in the article were showed here: CCL2-F:ATTCTGTGACCATCCCCTCAT,R:TGTATGTGCCTCTGAACCCAC;CCL3-F: TTCTCTGTACCATGACACTCTGC, R:CGTGGAATCTTCCGGCTGTAG; CCL4-F TTCCTGCTGTTTCTCTTACACCT, R: CTGTCTGCCTCTTTTGGTCAG; CCL5-F: GCTGCTTTGCCTACCTCTCC, R:TCGAGTGACAAACACGACTGC; CCL20-F: GCCTCTCGTACATACAGACGC, R:CCAGTTCTGCTTTGGATCAGC; VEGF-F: GAGGTCAAGGCTTTTGAAGGC, R:CTGTCCTGGTATTGAGGGTGG; PDGF-F: ACTTCTGTTGCTACACGAAGC, R:CGGTTGAGTCAGTGGAGTCC; IGF-F: AAATCAGCAGCCTTCCAACTC, R:GCACTTCCTCTACTTGTGTTCTT.

### WB detection

Mouse tissues and treated cells were lysed by RIPA (Solarbio® Life Science, R0010) that containing proteases and phosphorylases inhibitor cocktail. After BCA assay (Solarbio® Life Science, PC0020) for determining protein concentrations, 30 μg samples were loaded to a 12% SDS–polyacrylamide gel electrophoresis (SDS-PAGE) gel and then underwent the electrophoresis under 80 V for 120 min. The gel was transmitted to a methanol activated polyvinylidene fluoride (PVDF) (Millipore®, Meck, 0.45 um, IPVH00010) membranesand blocking by 5% BSA at 4℃ for 2 h. The next day, the PVDF membrane were cut into different strips according to molecular weight of different protein like MLKL (Cell Signaling Technology™, CST37705), RIPK3 (Cell Signaling Technology™, CST95702), p-MLKL(Cell Signaling Technology™, CST37333), p-RIPK3(Cell Signaling Technology™, CST93654), LC3b II (Abcam, ab192890), p62 (Abcam, ab109012), cleaved Casp3 (Cell Signaling Technology™, 9664), GAPDH (Servicebio®, GB15002), Actin (Servicebio®, GB111364), citrullinated Histone H3 (H3cit) (Abcam, ab219407), and so on at 4℃ overnight. The membranes were washed with TBST for three times and incubated with GAR/GAM-HRP for 2 h at room temperature. At last, the membranes were washed and stained by ECL chemiluminescence.

### RNA-sequencing and library construction

Human colonic epithelia cell NCM460 was subjected to treatment with 400 mM NaCl for 60 min or 500 mM sucrose for 90 min. Total RNA was isolated using Trizol reagent (Thermofisher, 15,596,018) according to the manufacturer's instructions.. The quality and quantity of total RNA were assessed using the Bioanalyzer 2100 and RNA 6000 Nano LabChip Kit (Agilent, CA, USA, 5067–1511). High-quality RNA samples with an RNA Integrity Number (RIN) number greater than 7.0 were used for constructing the sequencing library. After the extraction of RNA, mRNA was purified from total 5 μg of RNA using Dynabeads Oligo (dT) (Thermo Fisher, CA, USA) with two rounds of purification. Following purification, the mRNA was then fragmented into short fragments using divalent cations under elevated temperature (Magnesium RNA Fragmentation Module (NEB, cat. e6150, USA) at 94℃ for 5–7 min. The cleaved RNA fragments were reverse-transcribed using SuperScript™ II Reverse Transcriptase (Invitrogen, cat. 1,896,649, USA) to synthesize the complementary DNA (cDNA). The cDNA was further used for synthesizing U-labeled second-stranded DNAs with E. coli DNA polymerase I (NEB, cat. m0209, USA), RNase H (NEB, cat. m0297, USA) and dUTP Solution (Thermo Fisher, cat. R0133, USA). An A-base was added to the blunt ends of each strand to prepare them for ligation to the indexed adapters. The adapters were designed with a T-base overhang to facilitate ligation to the A-tailed fragmented DNA. Fragments were then ligated to the dual-index adapters were the, and size selection was performed with AMPureXP beads. Following treatment of the U-labeled second-stranded DNAs with the heat-labile UDG enzyme (NEB, cat.m0280, USA), the ligated products were amplified using PCR under the following conditions: initial denaturation at 95℃ for 3 min; 8 cycles of denaturation at 98℃ for 15 s, annealing at 60℃ for 15 s, and extension at 72℃ for 30 s; followed by final extension at 72℃ for 5 min. The final cDNA libraries had an average insert size of 300 ± 50 bp. Finally, paired-end sequencing was performed (2 × 150 bp) on an Illumina Novaseq™ 6000 (LC-Bio Technology CO., Ltd., Hangzhou, China) according to the vendor's recommended protocol.

### Bio-information analysis

The cDNA library was prepared using pooled RNA from HSD-treated NCM460 samples of human epithelial cell and sequenced on the Illumina Novaseq TM 6000splatform. The transcriptome was sequenced using the Illumina paired-end RNA-seq approach,, resulting in the generation of millions of 2 × 150 bp paired-end reads. The reads obtained from the sequencing machines may contain raw reads with adapters or low-quality bases, which can impact subsequent assembly and analysis process. To obtain high-quality clean reads, we performed additional filtering of the reads using Cutadapt (https://cutadapt.readthedocs.io/en/stable/, version: cutadapt-1.9). Gene function enrichment analysis was conducted using GO (Gene ontology) and KEGG (Kyoto Encyclopedia of Genes and Genomes) databases.

### ELISA assay

To identify the expression level of IL-33 in the mice model, whole blood was centrifugal at 12,000 rpm for 10 min, and then serum was separated and stored at -80℃ refrigerator. IL-33 coated antibodies were diluted and seeded into a 96-well ELISA (enzyme-linked immunosorbent assay) plate (Invitrogen™, BMS6025TEN). The plate was incubated overnight at 4℃, the next day, the supernatant was removed and the plate was washed with fresh PBS solution for three times. 300μL 5% Bovine serum albumin (BSA) were added into the plate for blocking for 2 h and then washed for three times. Mice serum was diluted and added to the plate followed by incubation at 4℃, the next day, removed the serum and washed three times. And the biotinylated IL-33 capture antibodies were added for 2 h at 37℃, and then coated with SAV-HRP. After incubation, the plate was washed three times and 100 μL TMB (3,3',5,5'-tetramethylbenzidine) solution was applied here. At last, 50 μL 2 M HCl was added to terminate the reaction and the OD values were recorded in the range of 408–600 nm.

### FCM assay

At the experiment endpoint, mice were sacrificed and spleens were collected for flow cytometry assay as previously described. Briefly, the spleen was filtered through a 200 mesh filter for single-cell preparation. After the removal of the suspension and centrifugal via lymphocyte separating fluid, the spleen lymphocytes were collected and washed with fresh PBS. 1 × 10^6^ lymphocytes were counted and seeded into 96-well plates, 1 μL different fluorescently labeled CD4/8/11b, F4/80, Gr-1 antibodies were added for staining 1 h. After washing with PBS, the lymphocytes were tested by Beckman Coulter CytoFLEX and analyzed via CytExpert.

### CCK-8 assay

1 × 10^5^ murine colonic epithelia cell CT26 was seeded to a 96-well cell plate and incubated at 37℃ overnight. The next day, 0, 50, 100, and 200 mM extra NaCl was added to analyze the role of high salt on colonic epithelial cells. After salt treatment, the Cell Counting Kit-8 (CCK-8) (Servicebio®, CA1210) assay kit was applied to test cell growth at 24, 48, and 96 h. Briefly, the cell supernatant was changed by 90 μL fresh medium and 10μL CCK8 solution was added to the cell culture medium. Then, the plate was incubated at 37℃ for 30 min and the OD_450nm_ value was recorded to measure the cell viability.

### MTT (3-[4,5-dimethylthiazol-2-yl]-2,5 diphenyl tetrazolium bromide) assay

1 × 10^5^ CT26 was seeded to a 96-well cell plate and incubated with 150, 200 mM NaCl with or without 100 ng/ml Lipopolysaccharides (LPS) (Servicebio®, L8880) pre-treatment. The next day, 10 μL MTT staining buffer (Servicebio®, M1020) was added to the cell and then incubated for 4 h. Then, 100 μL Formanzan lysis buffer was added and the plate was shacked for 10 min. After the crystals were dissolved, the OD_570 nm_ value was analyzed.

### ATP assay

1 × 10^6^ CT26 was seeded to a 12-well cell plate and incubated with 150, 200 mM NaCl with or without 100 ng/mL LPS pre-treatment. The next day, cell supernatants were removed and 200 μL adenosine triphosphate (ATP) lysis buffer was added (Servicebio®, BC0305). Then, cells was collected and centrifugal at 12, 000 g for 5 min. 20μL sample was mixed with 100 μL ATP working solution and the RLU value was calculated.

### Statistical analysis

All data was analyzed and drawn by Graphpad 8.0, student *t*-test was applied for two-group comparisons, one-way ANOVA was performed for three or more groups comparisons, and two-way ANOVA were used for survival rate comparisons. *p* value less than 0.05 was considered significant difference. * represented *p* < *0.05,* ** represented *p* < *0.01,* *** represented *p* < *0.001,* and **** represented *p* < *0.0001.*

### Supplementary Information

**Additional file 1: Supplement Figure1.** Sector Graph showed mapped region stat of each sample. **Supplement Figure 2.** Boxplot showed gene expression of all samples via log_10_(FPKM). **Supplement Figure 3.** The role of salt concentration on mice was showed here.

**Additional file 2: Supplement Table 1.** Reads QC information of NCM460 treated with salt or sucrose. **Supplement Table 2.** Mapped region stat information was supplied here.

## Data Availability

All data were listed in the article and raw data could be supplied when requested.
