# Supplementary material for: High-Salt-Diet (HSD) aggravates the progression of Inflammatory Bowel Disease (IBD) via regulating epithelial necroptosis
Source: Mol Biomed. 2023 Sep 11;4:28. doi: 10.1186/s43556-023-00135-1 (PMC10493205; doi:10.1186/s43556-023-00135-1)
Supplement: Supplementary file 1 — Additional file 1: Supplement Figure1. Sector Graph showed mapped region stat of each sample. Supplement Figure 2. Boxplot showed gene expression of all samples via log10(FPKM). Supplement Figure 3. The role of salt concentration on mice was showed here. [file 43556_2023_135_MOESM1_ESM.docx]

**Title Page**

**High-Salt-Diet (HSD) Aggravates the Progression of Inflammatory Bowel Disease (IBD) via Regulating Epithelial Necroptosis**

Jialong Qi^1,2*^, Jinli Wang^1,*^, Ying Zhang^1,3,*^, Huan Long^1,3^, Liang Dong^1,3^, Ping Wan^1,3^, ZanZuo^1,#^, Wenjie Chen^4,5#^, Zhengji Song^1#^

1. Yunnan Digestive Endoscopy Clinical Medical Center, Department of Gastroenterology, The First People's Hospital of Yunnan Province, Kunming 650032, P.R. China.
2. Yunnan Provincial Key Laboratory of Clinical Virology, The First People's Hospital of Yunnan Province, Kunming 650032, P.R. China.
3. School of Medicine, Kunming University of Science and Technology, Affiliated by The First People's Hospital of Yunnan Province, Kunming 650504, Yunnan, P.R. China;
4. State Key Laboratory of Respiratory Disease, Guangdong-Hongkong-Macao Joint Laboratory of Respiratory Infectious Disease, Guangzhou Medical University, Guangzhou, 510182, P.R. China;
5. Sydney Vital Translational Cancer Research Centre, Westbourne St, NSW 2065, Australia

^*^ co-first authors: Jialong Qi, Jinli Wang, and Ying Zhang

^#^Correspondence to: Zan Zuo [zuozan007@163.com](mailto:zuozan007@163.com), Zhengji Song [song4715@163.com](mailto:song4715@163.com), Wenjie Chen [wenjie.chen1@hdr.mq.edu.au](mailto:wenjie.chen1@hdr.mq.edu.au) <https://orcid.org/0000-0001-9512-9664>

**Graphical Abstract**

**
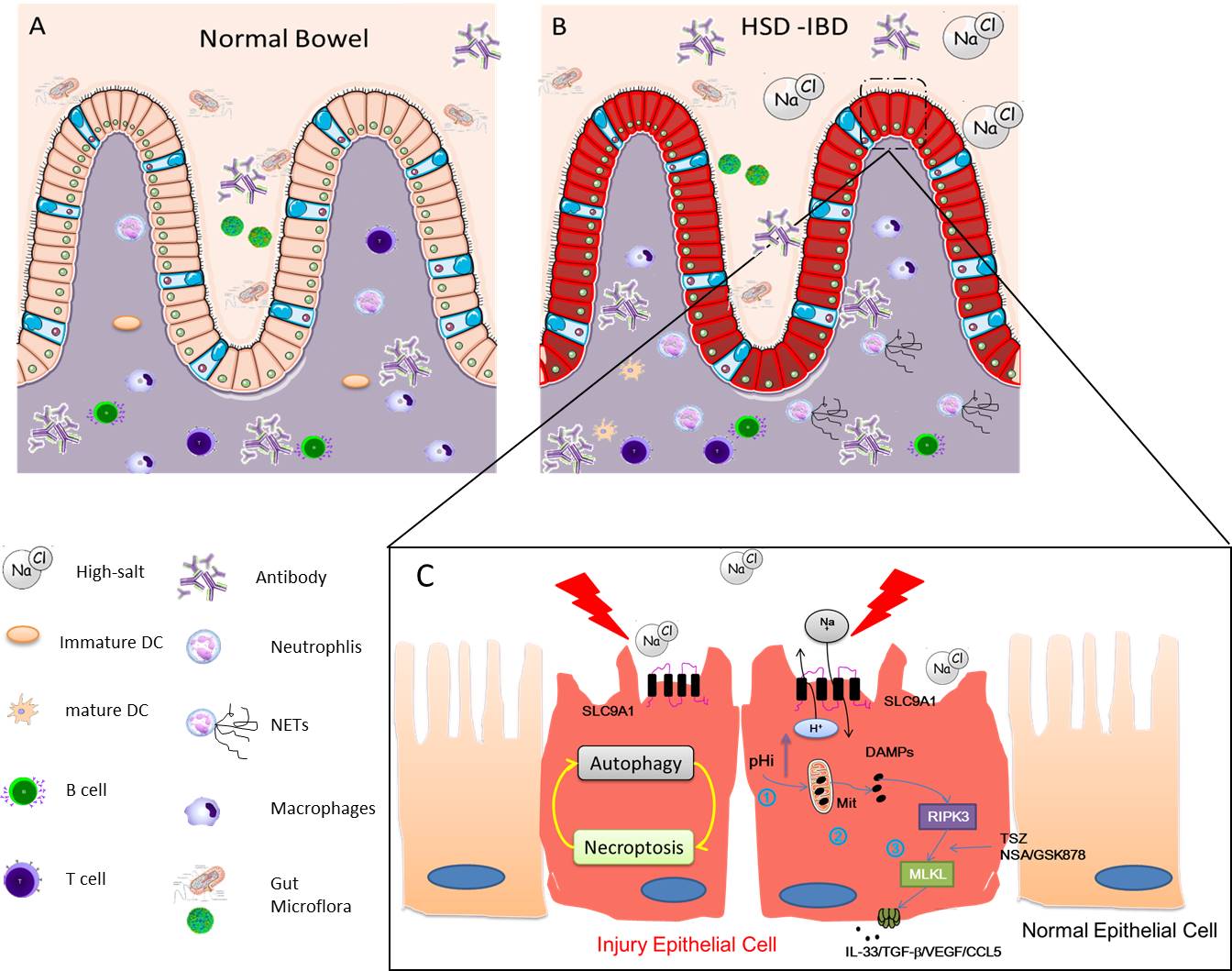
**

**Highlights**

1. High-salt and high-sucrose incubation enhanced RIPK3-dependent necroptosis in colonic epithelial cell.

2. HSD promotes DSS-induced IBD progression in a dose-dependent manner.

3. RIPK3 and MLKL genetic deletion significantly susceptible to IBD process.

**Supplementary** **Figure legends**

Supplement Figure1. Sector Graph showed mapped region stat of each sample.


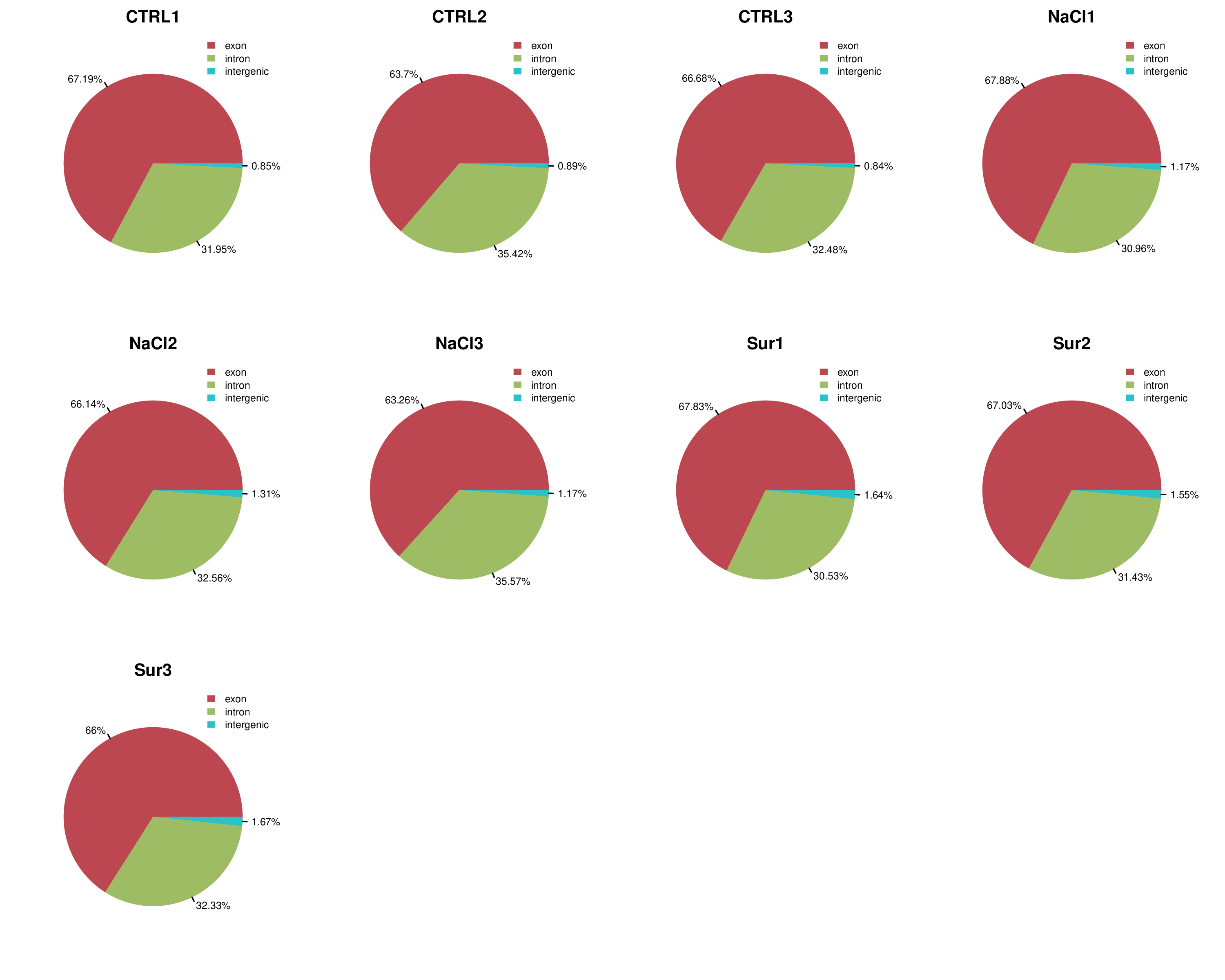


Supplement Figure 2. Boxplot showed gene expression of all samples via log_10_(FPKM).


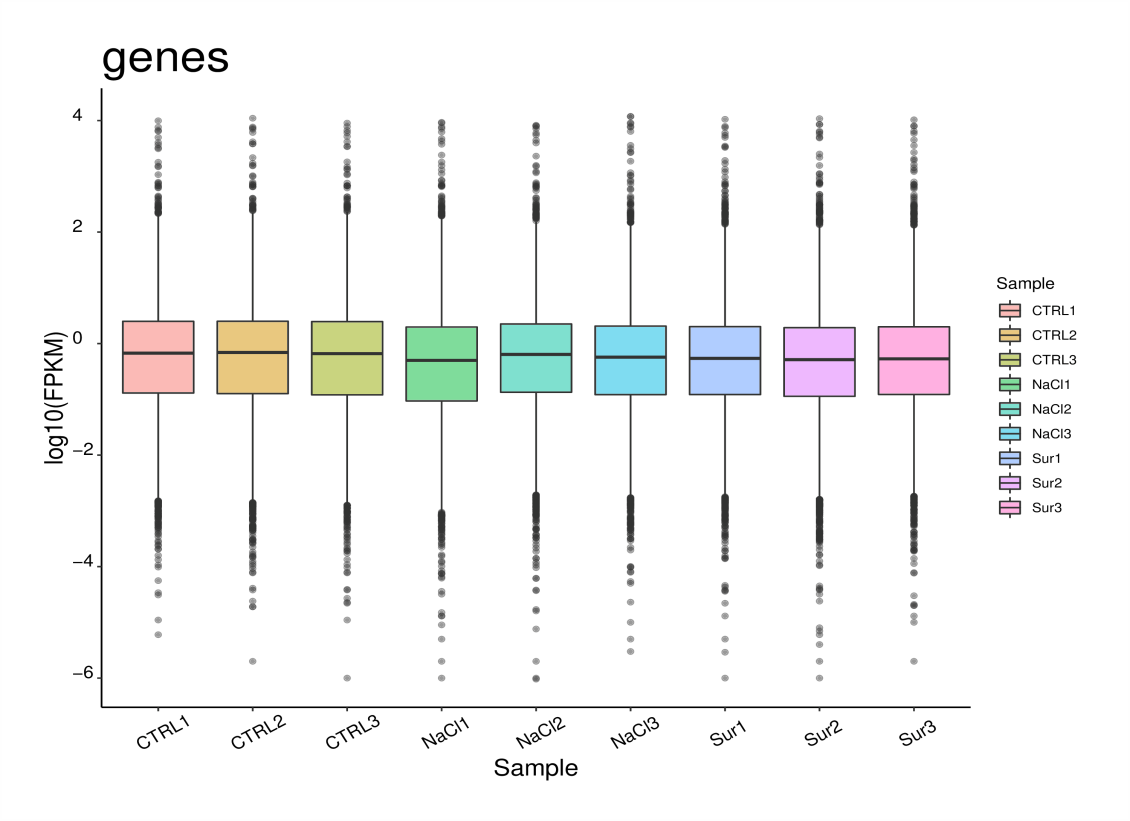


Supplement Figure 3. The role of salt concentration on mice was showed here.


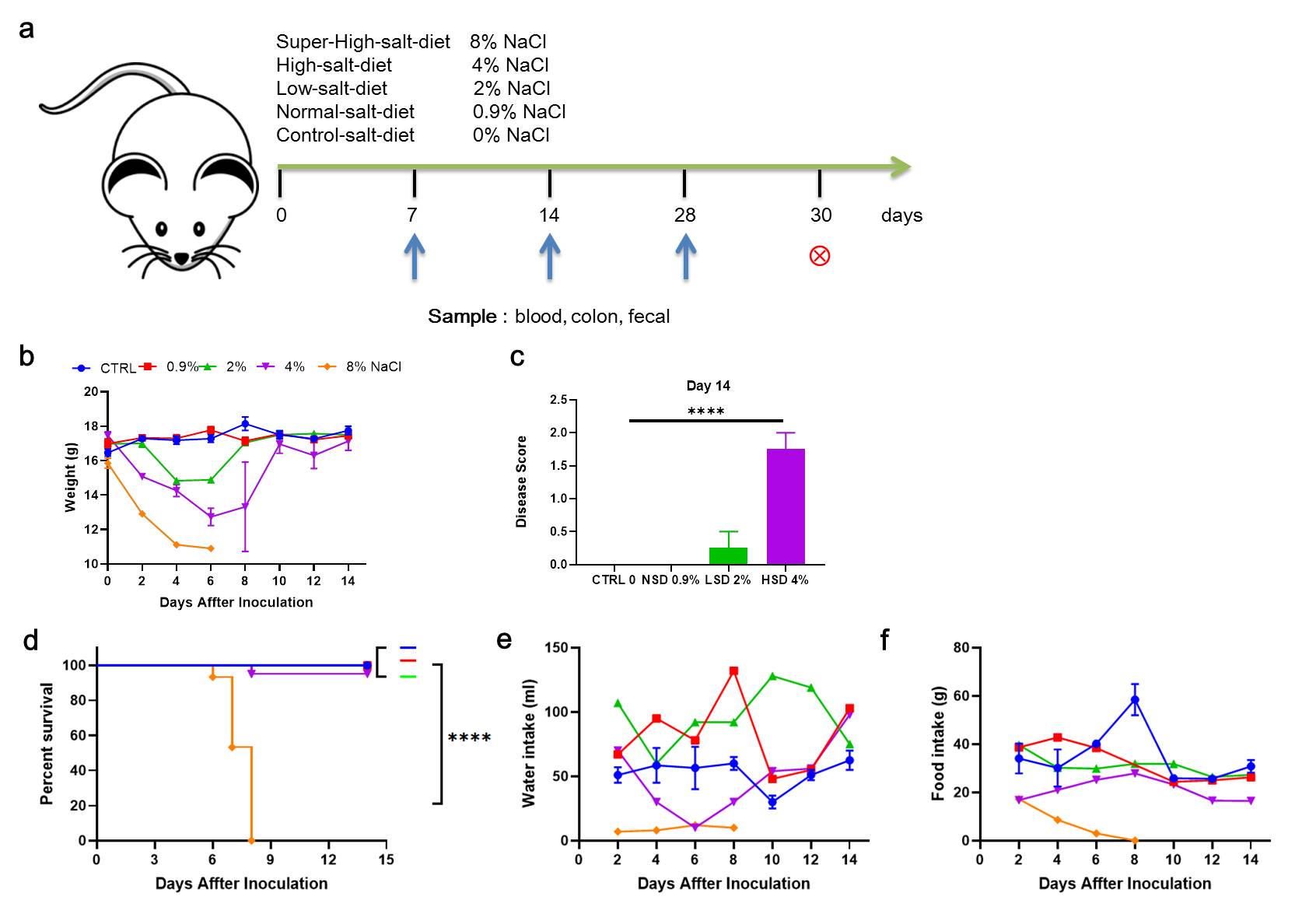


a. Schematic diagram of salt concentration on IBD progression. Mice were fed with different concentration salt-water, 0, 0.9, 2, 4, 8% were applied here. b. Body weight changes were showed here. c. Histogram showed mice diseases score at day 14. d. Survival curve of mice treated with different salt. e. water intake and f. Food intake of mice in each group.
